# Expression of the NS5 (VPg) Protein of Murine Norovirus Induces a G1/S Phase Arrest

**DOI:** 10.1371/journal.pone.0161582

**Published:** 2016-08-24

**Authors:** Colin Davies, Vernon K. Ward

**Affiliations:** Department of Microbiology and Immunology, Otago School of Medical Sciences, University of Otago, P. O. Box 56, Dunedin, 9054, New Zealand; Virginia Commonwealth University, UNITED STATES

## Abstract

Murine norovirus-1 (MNV-1) is known to subvert host cell division inducing an accumulation of cells in the G_0_/G_1_ phase, creating conditions where viral replication is favored. This study identified that NS5 (VPg), is capable of inducing cell cycle arrest in the absence of viral replication or other viral proteins in an analogous manner to MNV-1 infection. NS5 expression induced an accumulation of cells in the G_0_/G_1_ phase in an asynchronous population by inhibiting progression at the G_1_/S restriction point. Furthermore, NS5 expression resulted in a down-regulation of cyclin A expression in asynchronous cells and inhibited cyclin A expression in cells progressing from G_1_ to S phase. The activity of NS5 on the host cell cycle occurs through an uncharacterized function. Amino acid substitutions of NS5(Y26A) and NS5(F123A) that inhibit the ability for NS5 to attach to RNA and recruit host eukaryotic translation initiation factors, respectively, retained the ability to induce an accumulation of cells in the G_0_/G_1_ phase as identified for wild-type NS5. To the best of our knowledge, this is the first report of a VPg protein manipulating the host cell cycle.

## Introduction

Noroviruses are non-enveloped viruses from the *Caliciviridae* family that cause gastroenteritis in a variety of mammals including humans [1–5]. Human norovirus (HuNoV) infections account for significant mortality in the developing world, and in the developed world norovirus outbreaks come with a substantial financial burden [6]. HuNoV research has been hampered by the lack of a reproducible animal or cell culture system that supports viral replication. Using MNV-1 as a model allows norovirus replication and host cell interactions to be studied in cell culture and in small animals [7]. MNV-1 is a positive-sense RNA virus of approximately 7.4 kb, containing four open reading frames (ORF). ORF1 encodes for 6 non-structural proteins (NS1-2, NS3, NS4, NS5, NS6, and NS7) while ORF2 and ORF3 encode the major and minor structural proteins respectively [8]. ORF4 encodes for virulence factor 1, a non-essential protein involved in interactions with host apoptotic pathways [9].

The MNV NS5 (VPg; virus protein, genome linked), is a ~16 kDa protein that is covalently linked to the 5′ end of the genomic and subgenomic RNA [8]. Linkage to the genome is thought to prevent detection by host pathogen recognition receptors such as RIG-1 and protein kinase R that detect uncapped 5′ triphosphorylated RNA, leading to an antiviral response. NS5 additionally has a role in genome replication, acting in place of an RNA 5′ cap to provide a free hydroxyl that can be extended by the virally encoded RNA-dependent RNA polymerase (NS7) [10]. The NS5 protein also acts to aid viral translation, recruiting host eukaryotic translation initiation factors to initiate translation of viral proteins [11]. The NS5 protein also contains regions of predicted disorder that are often associated with multiple functions [12, 13].

As more viruses are characterized, it is becoming increasingly common to observe interactions between viral replication and the host cell cycle. Each phase of the cell cycle presents distinctive biological conditions that have a significant impact on viral replication. Many viruses can subvert the host cell division in order to create an environment where viral propagation is preferred. Several RNA viruses, including murine norovirus 1 (MNV-1) have been characterized to manipulate cell cycle progression at the G_1_/S restriction point, often creating favorable conditions for viral replication [14–21].

Cell cycle progression is a complex process that is tightly controlled by multiple pathways. The G_1_/S checkpoint controls progression from the first gap phase (G_1_), a period of substantial cell growth, into the synthesis phase (S) where the host DNA is replicated. Progression through G_1_/S is predominantly controlled by the phosphorylation status of the retinoblastoma protein (pRb), which is in turn controlled by the activities of cyclins and cyclin-dependent kinases (CDK) (reviewed in [22]). Cyclins are expressed at various stages of cell division and bind to their corresponding CDK and phosphorylate numerous targets including pRb. In early G_1_ phase, cyclin D family members bind to CDK4/6 and phosphorylate pRb, driving G_1_ phase progression and expression of E and A cyclins. Cyclin E forms a complex with CDK2, which further phosphorylates pRb to release an E2F transcription factor, driving S phase entry [23]. Cyclin A levels continue to increase during S phase and help drive cell cycle progression through the later stages of the cell cycle, through to the initiation of prophase during mitosis [24, 25].

Recently, we have shown that MNV-1 is able to manipulate the host cell division in murine macrophages, inducing an accumulation of cells in the G_0_/G_1_ phase due to an arrest at the G_1_/S restriction point [20]. Additionally, this G_1_/S arrest created conditions where MNV-1 replication was favored compared to other stages of the cell cycle. In this study, we show that expression of viral NS5 protein in cell culture induces an accumulation of cells in the G_0_/G_1_ phase through a G_1_/S arrest in an analogous manner to MNV-1 infection. Furthermore, the effects of NS5 on the host cell cycle are independent of the known replication and translation activities attributed to NS5 (VPg).

## Materials and Methods

### Cells

RAW-Blue cells, a mouse leukemic monocyte macrophage cell line (obtained from InvivoGen, San Diego, CA) were maintained in Dulbecco's modified Eagle's medium (DMEM) (Life Technologies, Gaithersburg, MD) containing 10% heat-inactivated fetal calf serum (Thermo Fisher Scientific), penicillin (100 U/ml), streptomycin (0.1 mg/ml), normocin (100 μg/ml) and zeocin (200 μg/ml) (Life Technologies). Cells were maintained at 37°C in a 5% CO_2_ humidified atmosphere and passaged every 48 h.

### Cloning and expression of NS1-2 and NS5

MNV-1 NS1-2 and NS5 sequences were amplified from the MNV-1 infectious clone pMNV*, derived from MNV strain CW1 (GenBank Accession DQ285629) as described in Ward et al [26], and the PCR products ligated into pUC8 and the sequence verified. Primers used for NS1-2 amplification were 5′ GAAATTAATACGACTCACTATAGTGAA**ATG**AGGATGGCAACGCCATC 3′ (forward) and 5′ AGCAAGGTCGAAGGG**TTA**TTCGGC 3′ (reverse) and for NS5 5′ GAAATTAATACGACTCACTATAGGGAGA**ATG**GGAAAGAAGGGCAAGA `3′ (reverse). Each forward primer added a GAAAT motif and a T7 promoter (underlined) to the 5′ end of the insert for T7 RNA polymerase binding as recommended for mMessage Machine *in vitro* RNA transcript production (Ambion). Bold sequences indicate start (ATG) or stop (TTA) codons. The expression constructs matched the MNV-1 NS1-2 and NS5 sequences except for the addition of a methionine codon at the 5′ end of the NS5 coding sequence to facilitate translation and stop codons in the NS1-2 and NS5 constructs to terminate translation. The resulting plasmids were transformed into XL1-Blue MRF′ *Escherichia coli* cells and used as templates for *in vitro* RNA synthesis.

#### Cloning of NS5(Y26A) and NS5(F123A)

Constructs for the expression of NS5 variants NS5(Y26A) and NS5(F123A) were designed with the requirements for RNA synthesis and translation and were synthesized by GenScript and cloned into a pUC57-Simple vector (GenScript, Piscataway, NJ, USA) [27]. The NS5 sequences (GenBank Accession DQ285629) were flanked at the 5′ end by a BamHI restriction site, a T7 promoter sequence (underlined), a Kozak sequence for optimal RNA translation and a methionine codon (bold) (GGATCCGAAATTAATACGACTCACTATAGGGAGA**ATG**). Flanking the 3′ end of the NS5 sequence was a stop codon (bold) and a HindIII restriction site (**TGA**AAGCTT). The NS5 variants sequences had alanine substitutions inserted at the tyrosine 26 and phenylalanine 123, named NS5(Y26A) and NS5(Y26A) respectively. The resulting plasmids were transformed into XL1-Blue MRF′ *E*. *coli* cells and used as templates for *in vitro* RNA synthesis.

#### *In vitro* RNA synthesis

Plasmids were linearized at the 3′ end of viral genes with EcoRI (for NS1-2), AvaI (for NS5) or HindIII (for NS5(Y26A) and NS5(F123A)). Subsequently, messenger RNA (mRNA) transcripts were synthesized from the linearized plasmids using the mMessage mMachine transcription kit (Ambion) and purified using MEGAclear transcription clean-up kit (Ambion).

#### Electroporation

Transfection of RNA transcripts was performed using a Neon Transfection system (Life Technologies), following manufacturer’s instructions. Briefly, RAW-Blue cells were suspended in resuspension buffer and approximately 1 × 10^6^ cells transfected with 4–6 μg of RNA using 1 pulse at 1730 V and 20 mA. Transfected cells were added to 2 ml of pre-warmed medium in a 6-well plate and incubated for the times indicated.

#### Western blot analysis

Transfected cells were collected posttransfection and washed twice in Dulbecco's phosphate buffered saline (dPBS). Cells were lysed directly in 25 μl dPBS and 25 μl sample buffer (120 mM Tris-HCl [pH 6.8], 5% SDS, 10% 2-mercaptoethanol, 20% glycerol, 0.01% bromophenol blue), boiled for 10 minutes and separated by SDS-PAGE electrophoresis. Proteins were transferred to nitrocellulose membranes (Amersham Hybond-C Extra; GE Healthcare) and detected with the corresponding primary and secondary antibodies. The following primary antibodies were used; cyclin A (H-432) (sc-751; Santa Cruz), actin (I-19) (sc-1616; Santa Cruz), GFP (ab6556; Abcam), MNV-1 anti-NS1-2 [13] and MNV-1 anti-NS5 [13]. Secondary antibodies used were 680RD donkey anti-goat IgG (926–68074; LI-COR) and 800CW donkey anti-rabbit IgG (926–32213; LI-COR).

### Cell Cycle Analysis

#### Synchronization of cells

To synchronize cells to the G_1_ phase, approximately 2 × 10^6^ cells were seeded in 25-cm^2^ flasks and treated with 3 mM sodium butyrate (*N*-butyrate) (B5887; Sigma) for 20 h [28, 29].

#### Cell cycle analysis by flow cytometry

Nuclear DNA content was measured by propidium iodide staining and fluorescence-activated cell sorting (FACS) as previously described [20]. Briefly, cells were scraped, washed in dPBS and fixed in 3 ml of cold 70% absolute ethanol overnight. Fixed cells were washed in dPBS and stained in 50 μg/ml propidium iodide (P4170; Sigma) and 0.1 mg/ml RNase A (R4875; Sigma) for 45 min at 37°C in 5% CO_2_. Stained cells were washed and analyzed using fluorescence-activated cell sorting (FACS); data was analyzed with MODfit LT 3.0 software (Verity Software House).

#### Statistical and densitometric analyses

Data is presented as means and standard deviations (SD). Results were analyzed with either a Student’s t-test or a one-way ANOVA with the appropriate post-test as stated. *P* values of ≤0.05 were considered statistically significant. Western blots are shown for one of three independent experiments. Band analysis for each protein was quantified using Image Studio Lite software. Each protein quantification was first normalized against actin loading before comparisons for changes (recorded as a percentage of mock-transfected) were made.

## Results

### Expression of NS5 induces a G_0_/G_1_ phase arrest

**Fig 1:**
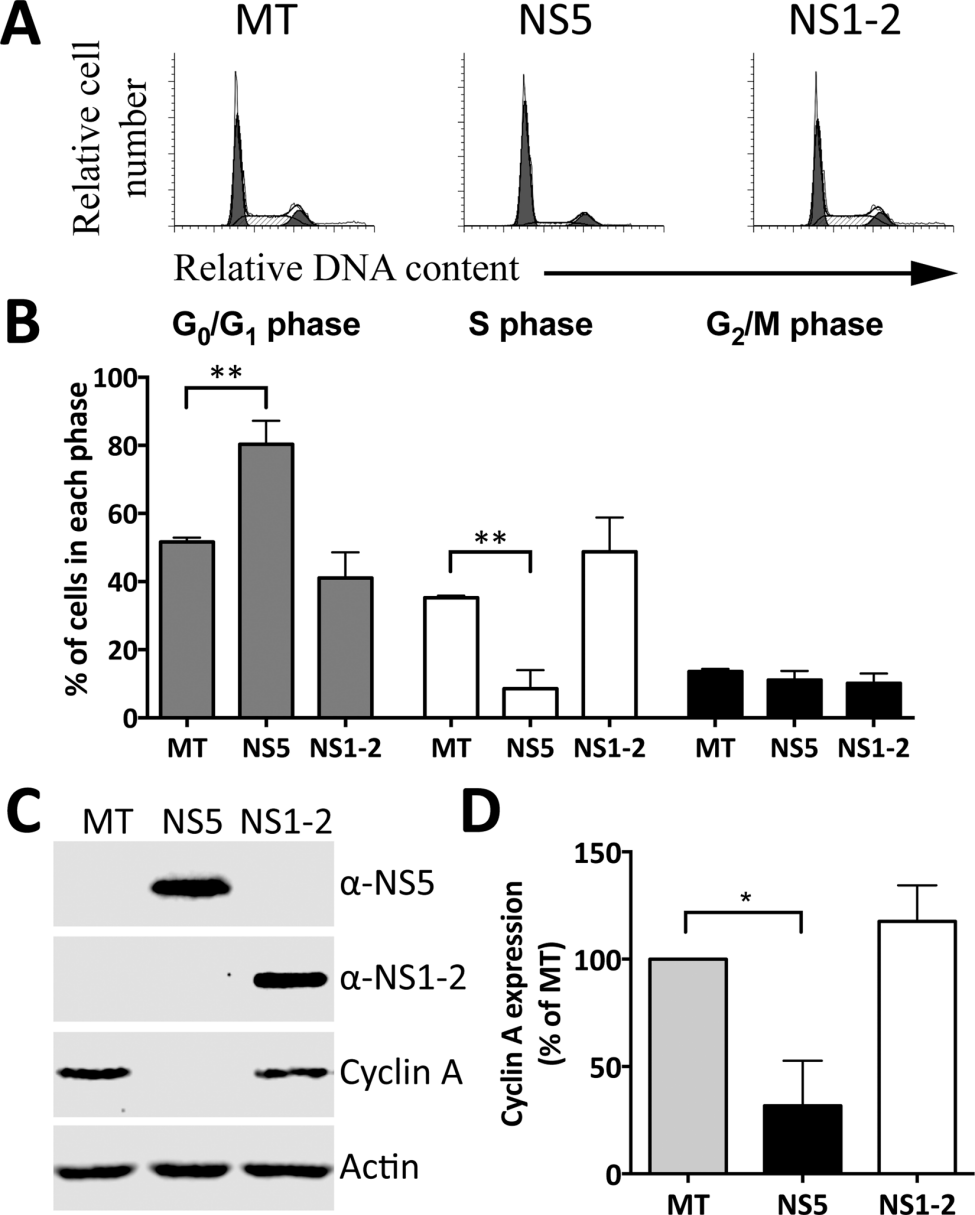
Expression of MNV-1 NS5 induces a G_0_/G_1_ phase arrest. Approximately 1 × 10^6^ RAW-Blue cells were transfected with 4–6 μg of NS1-2 or NS5 RNA transcripts. Mock-transfected (MT) cells were seeded at the time of transfection (negative control). (A) Cells were collected 18 hours posttransfection for FACS analysis of the cell cycle. The histograms presented are from one of three experiments. (B) The histograms from (A) were analysed with MODfit LT 3.0, and the percentage of cells in each phase of the cell cycle shown. Statistical significance was determined for comparisons between transfected populations and the corresponding mock-transfected population using a one-way ANOVA with Dunnett's post-test. **, *P* ≤ 0.01. (C) Expression of NS5, NS1-2 and cyclin A expression was determined by Western blot analysis. Actin was used as a loading control. (D) Cyclin A levels from three experiments were quantified with Image Studio Lite (LI-COR) and normalised against the results for actin and results presented as means and SD from three independent experiments. Statistical significance was determined for comparisons between transfected populations and the mock-transfected population using a one-sample t-test. *, *P* ≤ 0.05.

MNV-1 infection of RAW-Blue cells induces an arrest at the G_1_/S restriction point, increasing the G_0_/G_1_ population in order to change the internal cellular environment to favor viral replication [20]. We sought to determine if a viral encoded protein was responsible by analysis of the non-structural proteins of MNV-1 for effects to the host cell cycle. The NS5 protein from caliciviruses is essential to viral RNA transcription and translation and is essential for calicivirus replication. The NS5 protein has been shown to bind host eukaryotic translation initiation factors, recruiting these proteins for preferential viral translation and potentially inhibiting host protein expression [11, 30]. We hypothesized that an inhibition of host protein expression may contribute to a cell cycle arrest. The effect of NS5 on the host cell cycle was therefore determined by transfection of RAW-Blue cells with RNA transcripts, encoding individual viral genes, NS1-2 from MNV-1 was included as a negative control (Fig 1A). NS1-2 and NS5 were detected by their corresponding antibodies 18 h posttransfection (Fig 1C). Expression of NS5 increased the population of cells in the G_0_/G_1_ phase by 28% and decreased the S phase by 27% when compared to the mock-transfected population (Fig 1B). Furthermore, NS5 expression decreased cyclin A expression by 68% when compared to the mock-transfected control (Fig 1D). The cyclin A protein governs S phase entry and progression, so a decrease in expression would imply a decrease in S phase entry, further indicating NS5 as the protein responsible for the MNV-1 induced cell cycle manipulation. NS1-2 had no significant effects on the host cell cycle or cyclin A expression.

### NS5 inhibits cyclin A expression and induces an arrest at the G_1_/S restriction point

**Fig 2:**
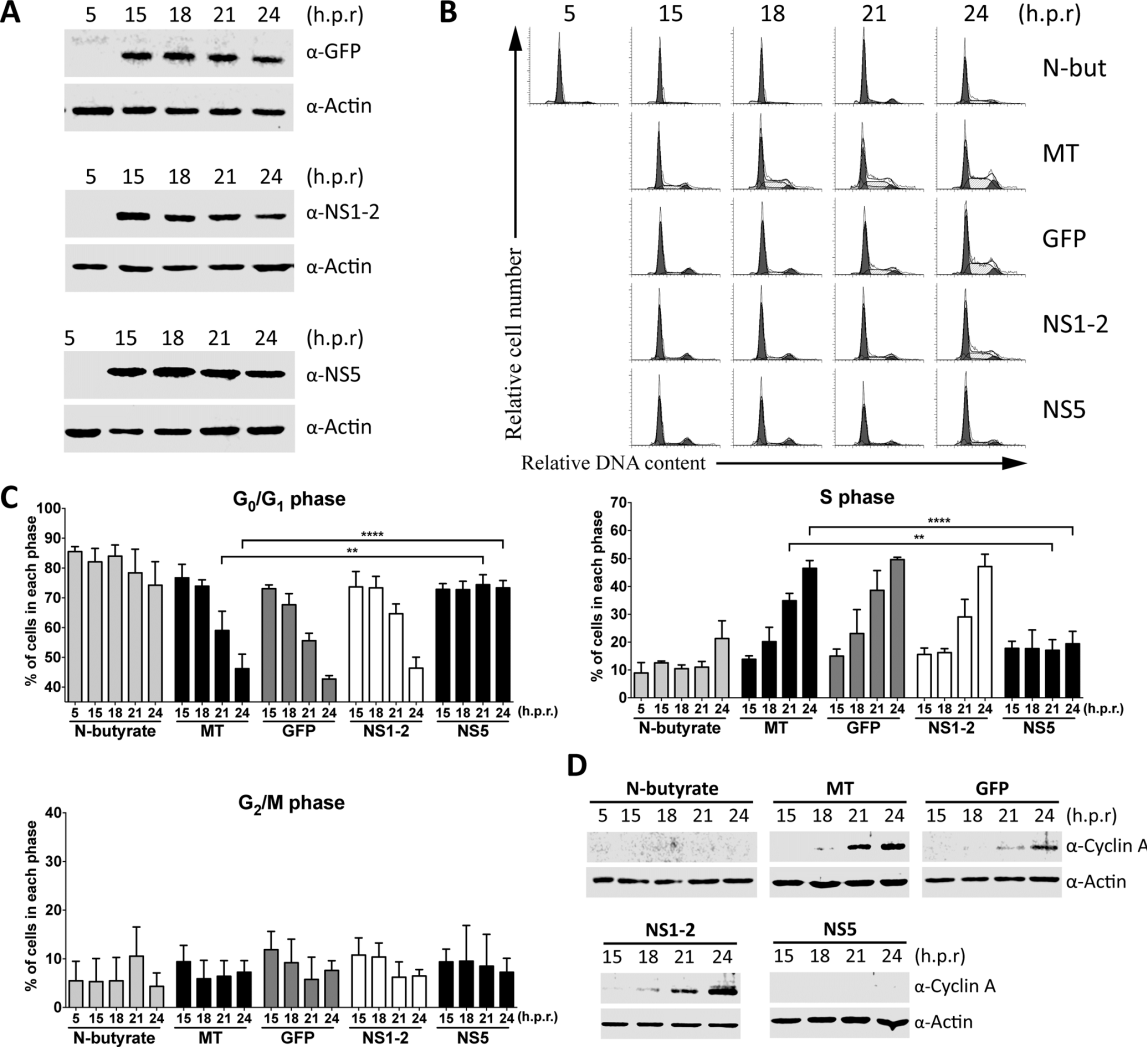
The MNV-1 protein NS5 induces a G_1_/S arrest and inhibits cyclin A expression. Approximately 7.5 × 10^6^ RAW-Blue cells were seeded in 75 cm^2^ flasks and treated with 3 mM *N*-butyrate for 20 h to synchronize cells into the G_1_ phase. Following synchronization, cells were washed 3 times with 5 ml of complete medium and incubated for 5 h. After incubation, cells were recovered, counted and approximately 1 × 10^6^ cells transfected with 4–6 μg of GFP, NS1-2 or NS5 RNA transcripts. A population of cells had 3 mM of *N*-butyrate added at the time of transfection (positive control) and mock-transfected (MT) cells were seeded at the time of transfection (negative control). (A) At the indicated h post-release (h.p.r.) cells were harvested for Western blot analysis of viral protein expression. The blots presented are representative blots from three experiments. (B) Cells were collected post-release at the indicated times for FACS analysis of the cell cycle. The histograms presented are from one of three experiments. (C) The histograms from (B) were analyzed with MODfit LT 3.0, and the percentage of cells in each phase of the cell cycle shown. The results are means and SD from three independent experiments. Statistical significance was determined for comparisons between transfected populations and the corresponding mock-transfected time point using a one-way ANOVA with Dunnett's post-test. **, *P* ≤ 0.01; ***, *P* ≤ 0.001; ****, *P* ≤ 0.0001. (D) Cells were harvested at the indicated h.p.r. for Western blot analysis of cyclin A and actin expression. The blot presented is a representative blot from three experiments.

If the NS5 protein is responsible for the cell cycle arrest, then it should induce its cell cycle arrest at the G_1_/S restriction point, as observed during MNV-1 infections [20]. The transition through G_1_/S is a highly regulated checkpoint during cell division and is often targeted by viruses to induce changes to the host cell cycle [14, 18, 31, 32]. The transition of cells through the G_1_/S restriction point was examined in cells expressing NS5. Cells were synchronized to the G_1_ phase through *N*-butyrate treatment, released from the arrest and transfected with NS5, NS1-2 and GFP coding RNA. NS1-2 was used as a viral control protein that did not affect the cell cycle and GFP as a non-viral RNA negative control. *N*-butyrate was also added to released populations and used as a positive control. Cells were then analyzed for their transition from G_1_ into S phase by FACS analysis of the host cell cycle. GFP, NS1-2 and NS5 RNA transcripts were translated into protein and detected from 15 to 24 hours post-G_1_ release (Fig 2A). The *N*-butyrate treated cells remained predominantly in G_1_ phase post-release while the mock-transfected cells came out of the G_1_ arrest, indicated by a decrease in the G_0_/G_1_ population and an increase in the S phase cells post-release (Fig 2C). Transfection of GFP and NS1-2 RNA had no effect on the transition of cells from G_1_ into S phase compared to the mock-transfected population, with both GFP and NS1-2 transfected populations progressing into S phase, with a substantial decrease in the G_0_/G_1_ population observed at 21 hours post-release, similar to the mock-transfected population (Fig 2C). In contrast, transfection of NS5 RNA induced a G_1_/S phase arrest, as indicated by cells remaining in the G_1_ phase post-release, with no increase in the S phase population. At 24 hours post-release in NS5 transfected populations, 73% of cells remained in the G_0_/G_1_ phase compared to 46% in the mock-transfected population (Fig 2C).

Because cyclin A expression is inhibited by MNV-1 infection in cells transitioning through the G_1_/S restriction point [20], cells were analyzed post-G_1_ release for cyclin A expression by Western blot analysis. Expression of NS5 inhibited the accumulation of cyclin A in populations progressing from G_1_ to S phase. In a G_1_ arrested population expression of cyclin A is low, indicated by undetectable levels of cyclin A in *N*-butyrate treated cells (Fig 2D). In mock-transfected, GFP and NS1-2 transfected populations, cyclin A expression increased from 18 to 24 hours post-release as cells entered S phase. In NS5 transfected cells, cyclin A levels remained below detectable limits post-release, as transfected cells remained in G_0_/G_1_ phase and did not progress into S phase. These results indicate that NS5 is responsible the cell cycle arrest induced by MNV-1. Not only does NS5 expression increase the G_0_/G_1_ population, but it also inhibits progression at the G_1_/S restriction point and inhibits cyclin A expression.

### NS5 association with host eukaryotic initiation factors does not influence NS5 induced cell cycle arrest

**Fig 3:**
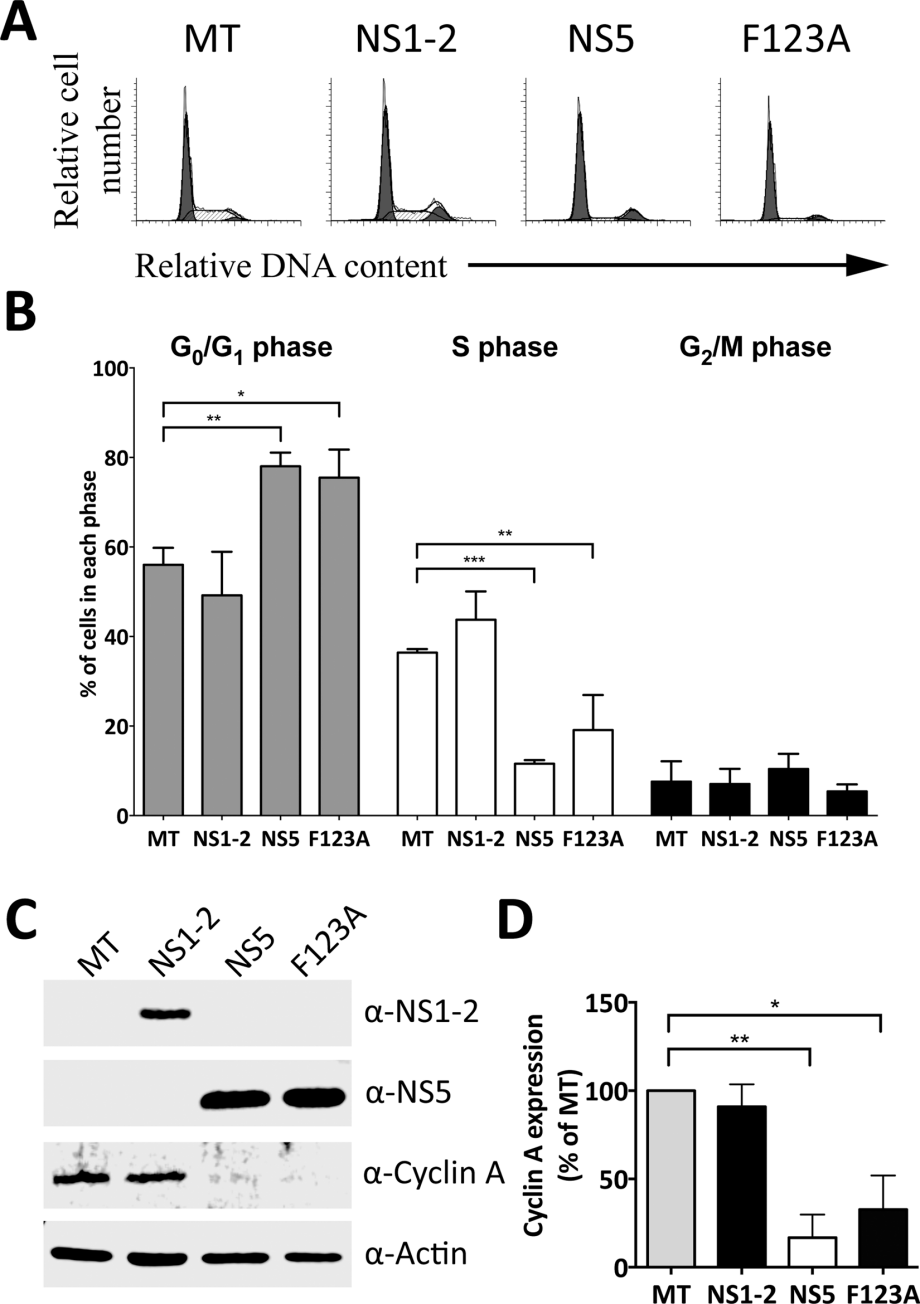
The eukaryotic initiation factor binding domain is not responsible for the NS5 induced cell cycle effects. Approximately 1 × 10^6^ RAW-Blue cells were transfected with 4–6 μg of NS1-2, WT NS5 and NS5(F123A) RNA transcripts. Mock-transfected (MT) cells were seeded at the time of transfection (negative control). (A) Cells were collected 18 hours posttransfection for FACS analysis of the cell cycle. The histograms presented are from one of three experiments. (B) The histograms from (A) were analysed with MODfit LT 3.0, and the percentage of cells in each phase of the cell cycle shown. Statistical significance was determined for comparisons between transfected populations and the corresponding mock-transfected population using a one-way ANOVA with Dunnett's post-test. *, *P* ≤ 0.05; **, *P* ≤ 0.01; ***, *P* ≤ 0.001. (C) Expression of NS1-2, WT NS5, NS5(F123A) and Cyclin A was determined by Western blot analysis, actin was used as a loading control. (D) Cyclin A levels from three experiments were quantified with Image Studio Lite (LI-COR) and normalised against the results for actin and results presented as means and SD from three independent experiments. Statistical significance was determined for comparisons between transfected populations and the mock-transfected population using a one-sample t-test. *, *P* ≤ 0.05; **, *P* ≤ 0.01.

A well-characterized function of NS5 in viral replication is to recruit host eukaryotic initiation factors for preferential translation of viral proteins [30, 33]. It was hypothesized that the manipulation of the host cell cycle by NS5 could be driven by its association with host eukaryotic initiation factor eIF4G, leading to inhibition of host translation, as seen with plant VPg proteins [34], and thus potentially inducing a cell cycle arrest. The ability of MNV NS5 to bind to eIF4G can be abolished through the introduction of a phenylalanine to alanine substitution at position 123 (NS5(F123A)) [30]. Not only does the NS5(F123A) substitution inhibit binding to scaffold protein eIF4G, it abolishes viral replication. We predicted that the introduction of the NS5(F123A) substitution could inhibit its cell cycle control. RNA transcripts encoding WT NS5, NS5(F123A) and NS1-2 were generated, transfected into an asynchronous cell population and their cell cycle effects analyzed by flow cytometry. Both NS5 and NS5(F123A) could be detected by the α-NS5 antibody (Fig 3C). Expression of viral NS1-2 had no effect on the host cell cycle while expression of both NS5 and the NS5(F123A) variant increased the G_0_/G_1_ population by ~22% and decreased the S phase population proportionally when compared to the mock-transfected population (Fig 3B). Furthermore, the NS5(F123A) variant decreased cyclin A protein expression by 67% when compared to the mock-transfected population in a synonymous manner to NS5, strongly implying that the host eukaryotic initiation factor binding domain of NS5 does not play a role in its cell cycle manipulation (Fig 3D).

### NS5 nucleotidylation to RNA via Y26 plays no role in inducing host cell cycle effects

**Fig 4:**
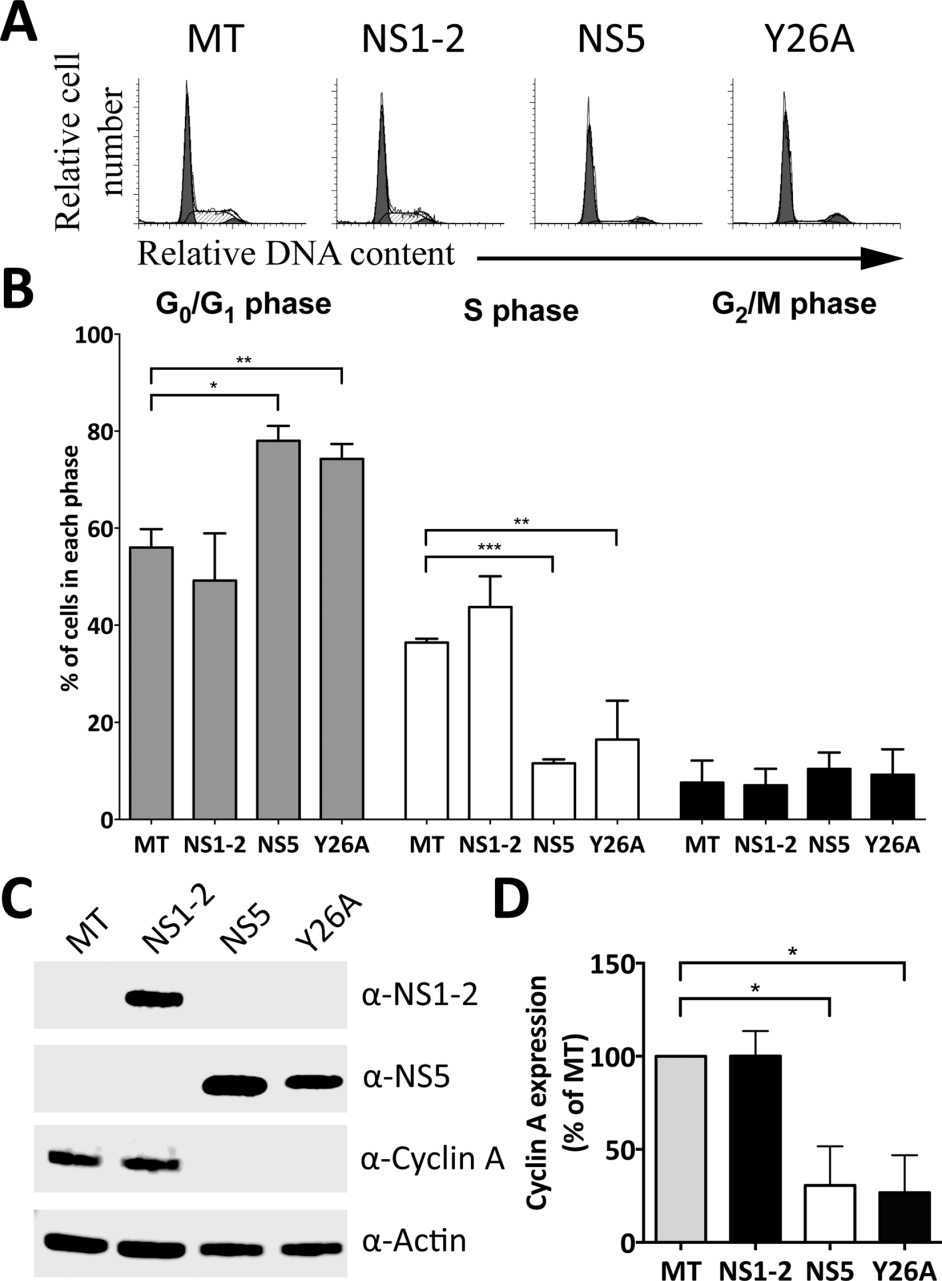
Nucleotidylation via Y26 is not responsible for the NS5 induced cell cycle effects. Approximately 1 × 10^6^ RAW-Blue cells were transfected with 4–6 μg of NS1-2, WT NS5 and NS5(Y26A) RNA transcripts. A mock-transfected (MT) control was seeded at the time of transfection (negative control). (A) Cells were collected 18 hours posttransfection for FACS analysis of the cell cycle. The data are from one of three experiments. (B) The histograms from (A) were analysed with MODfit LT 3.0, and the percentage of cells in each phase of the cell cycle shown. Statistical significance was determined for comparisons between transfected populations and the corresponding mock-transfected population using a one-way ANOVA with Dunnett's post-test. *, *P* ≤ 0.05; **, *P* ≤ 0.01; ***, *P* ≤ 0.001. (C) Expression of NS1-2, WT NS5, NS5(Y26A) and cyclin A was determined by Western blot analysis. Actin was used as a loading control. (D) Cyclin A levels from three experiments were quantified with Image Studio Lite (LI-COR) and normalised against the results for actin. The results are presented as means and SD from three independent experiments. Statistical significance was determined for comparisons between transfected populations and the mock-transfected population using a one-sample t-test. *, *P* ≤ 0.05.

The NS5 protein from MNV is covalently attached at the 5′ terminus of viral RNA, acting as a cap to prime RNA synthesis [8]. Attachment of NS5 to viral RNA occurs via the tyrosine residue at position 26 (Y26) in MNV, lying within a highly conserved EYDE motif in caliciviruses [10, 35, 36]. Substitution of the Y26 residue with an alanine residue NS5(Y26A) prevents the formation of NS5-viral RNA [10, 37]. The nucleotidylation of NS5 at Y26 is likely contingent upon viral RNA polymerase (NS7), which is not present in the expression system used in this study. To further confirm that Y26 residue is not involved in the cell cycle arrest through an unidentified mechanism, an alanine substitution at Y26 was introduced (NS5(Y26)). Although the viral genome and NS7 protein is absent in the transfection, we could not exclude that NS5 could be attaching via the Y26 residue to host RNA. RNA encoding WT NS5, NS5(Y26A) and NS1-2 were synthesized, transfected into an asynchronous cell population and their cell cycle effects analyzed by flow cytometry. Both NS5 and NS5(Y26A) could be detected by the α-NS5 antibody (Fig 4C). Expression of viral NS1-2 had no effect on the host cell cycle while both NS5 and NS5(Y26A) expression increased the G_0_/G_1_ population by ~20% and decreased the S phase population proportionally when compared to the mock-transfected population (Fig 4B). Furthermore, WT NS5 and NS5(Y26A) decreased cyclin A protein expression by 69% and 73% respectively when compared to the mock-transfected population (Fig 4D). These results, combined with the absence of viral RNA polymerase, imply that nucleotidylation to RNA via the Y26 residue has no role in NS5 ability to manipulate the host cell cycle.

## Discussion

We recently identified that MNV-1 was able to manipulate the host cell cycle, causing an arrest and accumulation in an early cell growth phase leading to enhanced viral replication [20]. This study identifies that the NS5 protein from MNV-1 is able to induce a cell cycle arrest analogous to that of MNV-1 infection in a mouse macrophage cell line, in the absence of other viral factors. Changes to the host cell cycle post NS5 expression was nearly identical to that observed under MNV-1 infection with the G_0_/G_1_ population increasing by 24% and 28% and the corresponding S phase population decreasing by 26% and 27% respectively. Expression of NS5 alone was sufficient to induce an accumulation of cells in the G_0_/G_1_ phase and reduce cyclin A expression in an asynchronous population. Further confirmation of NS5 as the cell cycle regulator comes from analyzing the ability of NS5 to inhibit the G_1_/S transition and prevent cyclin A accumulating in a population progressing from G_1_ into S phase. These effects on the host cell cycle are consistent with the observed effects of MNV-1 infection, showing NS5 to be a causative agent of this cell cycle arrest.

It was initially hypothesized that the cell cycle arrest induced by NS5 was due to its known interaction with host eIF4G. The NS5 protein from several caliciviruses has been documented to aid in viral protein translation, through binding to several host eukaryotic initiation factors and recruiting ribosomes to the site of viral replication [11, 30, 38–40]. The concentration of host eukaryotic initiation factors by VPg proteins is predicted to impair host translation [36]. Shut-off of host protein translation has been shown to induce cell cycle arrests in several cell phases including G_1_ and G_2_/M [41, 42]. We analyzed the impact on the host cell cycle of NS5 binding to host eukaryotic initiation factors through the introduction of an amino acid substitution that was known to impede binding to eIF4G [30]. The NS5(F123A) substitution had no effect on the ability of NS5 to induce a G_1_ arrest indicating an alternative mechanism is responsible for the cell cycle manipulation.

Another well characterized function of NS5 is in priming of RNA synthesis via attachment to viral genomic and subgenomic RNA. While nucleotidylation of the NS5 Y26 residue is unlikely in the absence of viral RNA polymerase (NS7) we wanted to confirm that Y26 had no role in cell cycle arrest. Attachment to host RNA could result in changes in protein expression, leading to a cell cycle arrest. The NS5(Y26A) protein is unable to undergo the nucleotidylation reaction that attaches NS5 to viral RNA but still induced cell cycle changes in an analogous manner to WT NS5. This indicates that attachment to RNA via the Y26 residue is not the cause of cell cycle affects. MNV NS5 is indicated to be located in the perinuclear region, so an interaction with host DNA is unlikely [43]. Because the NS5(Y26A) and NS5(F123A) substitutions could still induce cell cycle control suggests that the NS5 induced G_1_/S arrest occurs through an as yet uncharacterized activity.

In this study, our data discovers a viral protein that independent of other viral proteins is able to induce cell cycle manipulation and identifies a new function of the multi-faceted NS5 protein. The detailed mechanism of how NS5 is inhibiting cell cycle progression may lie with the observed decrease in cyclin A expression with further studies needed to examine this interaction. Nevertheless, these results reinforce the importance of the multifunctional NS5 in norovirus replication.

## References

[1] Xi JN, Graham DY, Wang KN, Estes MK. Norwalk virus genome cloning and characterization. Science. 1990; 250:1580–1583. 2177224 10.1126/science.2177224

[2] Hansman GS, Oka T, Katayama K, Takeda N. Human sapoviruses: genetic diversity, recombination, and classification. Rev Med Virol. 2007; 17:133–141. 17340567 10.1002/rmv.533

[3] Bergin IL, Wise AG, Bolin SR, Mullaney TP, Kiupel M, Maes RK. Novel calicivirus identified in rabbits, Michigan, USA. Emerg Infect Dis. 2009; 15:1955–62. 10.3201/eid1512.090839 19961675 PMC3044539

[4] Radford AD, Addie D, Belak S, Boucraut-Baralon C, Egberink H, Frymus T, et al Feline calicivirus infection. ABCD guidelines on prevention and management. J Feline Med Surg. 2009; 11:556–564. 10.1016/j.jfms.2009.05.004 19481035 PMC11132273

[5] Karst SM, Wobus CE, Lay M, Davidson J, Virgin HWt. STAT1-dependent innate immunity to a Norwalk-like virus. Science. 2003; 299:1575–1578. 12624267 10.1126/science.1077905

[6] Lozano R, Naghavi M, Foreman K, Lim S, Shibuya K, Aboyans V, et al Global and regional mortality from 235 causes of death for 20 age groups in 1990 and 2010: a systematic analysis for the Global Burden of Disease Study 2010. Lancet. 2012; 380:2095–2128. 10.1016/S0140-6736(12)61728-0 23245604 PMC10790329

[7] Wobus CE, Karst SM, Thackray LB, Chang KO, Sosnovtsev SV, Belliot G, et al Replication of Norovirus in cell culture reveals a tropism for dendritic cells and macrophages. PLoS Biol. 2004; 2:e432 15562321 10.1371/journal.pbio.0020432 PMC532393

[8] Sosnovtsev SV, Belliot G, Chang KO, Prikhodko VG, Thackray LB, Wobus CE, et al Cleavage map and proteolytic processing of the murine norovirus nonstructural polyprotein in infected cells. J Virol. 2006; 80:7816–7831. 16873239 10.1128/JVI.00532-06 PMC1563789

[9] McFadden N, Bailey D, Carrara G, Benson A, Chaudhry Y, Shortland A, et al Norovirus regulation of the innate immune response and apoptosis occurs via the product of the alternative open reading frame 4. PLoS Pathog. 2011; 7:e1002413 10.1371/journal.ppat.1002413 22174679 PMC3234229

[10] Subba-Reddy CV, Goodfellow I, Kao CC. VPg-primed RNA synthesis of norovirus RNA-dependent RNA polymerases by using a novel cell-based assay. J Virol. 2011; 85:13027–13037. 10.1128/JVI.06191-11 21994457 PMC3233154

[11] Chaudhry Y, Nayak A, Bordeleau ME, Tanaka J, Pelletier J, Belsham GJ, et al Caliciviruses differ in their functional requirements for eIF4F components. The J Biol Chem. 2006; 281:25315–25325. 16835235 10.1074/jbc.M602230200

[12] Hebrard E, Bessin Y, Michon T, Longhi S, Uversky VN, Delalande F, et al Intrinsic disorder in Viral Proteins Genome-Linked: experimental and predictive analyses. Virol J. 2009; 6:23 10.1186/1743-422X-6-23 19220875 PMC2649914

[13] Baker E. Characterisation of the NS1-2 and NS4 proteins of murine norovirus: PhD Thesis. University of Otago, Microbiology & Immunology; 2012.

[14] He Y, Xu K, Keiner B, Zhou J, Czudai V, Li T, et al Influenza A virus replication induces cell cycle arrest in G0/G1 phase. J Virol. 2010; 84:12832–12840. 10.1128/JVI.01216-10 20861262 PMC3004346

[15] Gibbs JD, Ornoff DM, Igo HA, Zeng JY, Imani F. Cell cycle arrest by transforming growth factor beta1 enhances replication of respiratory syncytial virus in lung epithelial cells. J Virol. 2009; 83:12424–12431. 10.1128/JVI.00806-09 19759128 PMC2786720

[16] Naniche D, Reed SI, Oldstone MB. Cell cycle arrest during measles virus infection: a G0-like block leads to suppression of retinoblastoma protein expression. J Virol. 1999; 73:1894–1901. 9971768 10.1128/jvi.73.3.1894-1901.1999 PMC104430

[17] Feuer R, Mena I, Pagarigan R, Slifka MK, Whitton JL. Cell cycle status affects coxsackievirus replication, persistence, and reactivation in vitro. J Virol. 2002; 76:4430–4440. 11932410 10.1128/JVI.76.9.4430-4440.2002 PMC155066

[18] Chen CJ, Makino S. Murine coronavirus replication induces cell cycle arrest in G0/G1 phase. J Virol. 2004; 78:5658–5669. 15140963 10.1128/JVI.78.11.5658-5669.2004 PMC415820

[19] Surjit M, Liu B, Chow VT, Lal SK. The nucleocapsid protein of severe acute respiratory syndrome-coronavirus inhibits the activity of cyclin-cyclin-dependent kinase complex and blocks S phase progression in mammalian cells. J Biol Chem. 2006; 281:10669–81. 16431923 10.1074/jbc.M509233200 PMC7995956

[20] Davies C, Brown C, Westphal D, Ward J, Ward VK. Murine Norovirus replication induces a G0/G1 cell cycle arrest in asynchronous growing cells. J Virol. 2015; 89:6057–6066. 10.1128/JVI.03673-14 25810556 PMC4442456

[21] Satyanarayana A, Kaldis P. Mammalian cell-cycle regulation: several Cdks, numerous cyclins and diverse compensatory mechanisms. Oncogene. 2009; 28:2925–2939. 10.1038/onc.2009.170 19561645

[22] Obaya AJ, Sedivy JM. Regulation of cyclin-Cdk activity in mammalian cells. Cell Mol Life Sci. 2002; 59:126–142. 11846025 10.1007/s00018-002-8410-1 PMC11337483

[23] Lundberg AS, Weinberg RA. Functional inactivation of the retinoblastoma protein requires sequential modification by at least two distinct cyclin-cdk complexes. Mol Cell Biol. 1998; 18:753–761. 9447971 10.1128/mcb.18.2.753 PMC108786

[24] Furuno N, den Elzen N, Pines J. Human cyclin A is required for mitosis until mid prophase. J Cell Biol. 1999; 147(2):295–306. 10525536 10.1083/jcb.147.2.295 PMC2174228

[25] Coverley D, Laman H, Laskey RA. Distinct roles for cyclins E and A during DNA replication complex assembly and activation. Nat Cell Biol. 2002; 4:523–528. 12080347 10.1038/ncb813

[26] Ward VK, McCormick CJ, Clarke IN, Salim O, Wobus CE, Thackray LB, et al Recovery of infectious murine norovirus using pol II-driven expression of full-length cDNA. Proc Natl Acad Sci U S A. 2007; 104:11050–11055. 17581883 10.1073/pnas.0700336104 PMC1904157

[27] Guo X, Zhang T, Hu Z, Zhang Y, Shi Z, Wang Q, et al Efficient RNA/Cas9-mediated genome editing in Xenopus tropicalis. Development. 2014; 141:707–714. 10.1242/dev.099853 24401372

[28] Kruh J, Defer N, Tichonicky L. [Molecular and cellular action of butyrate]. C R Seances Soc Biol Fil. 1992; 186:12–25. 1450986

[29] Golzio M, Teissie J, Rols MP. Cell synchronization effect on mammalian cell permeabilization and gene delivery by electric field. Biochim Biophys Acta. 2002; 1563:23–28. 12007621 10.1016/s0005-2736(02)00369-3

[30] Chung L, Bailey D, Leen EN, Emmott EP, Chaudhry Y, Roberts LO, et al Norovirus translation requires an interaction between the C Terminus of the genome-linked viral protein VPg and eukaryotic translation initiation factor 4G. J Biol Chem. 2014; 289:21738–21750. 10.1074/jbc.M114.550657 24928504 PMC4118132

[31] Bresnahan WA, Boldogh I, Thompson EA, Albrecht T. Human cytomegalovirus inhibits cellular DNA synthesis and arrests productively infected cells in late G1. Virology. 1996; 224:150–160. 8862409 10.1006/viro.1996.0516

[32] Ehmann GL, McLean TI, Bachenheimer SL. Herpes simplex virus type 1 infection imposes a G(1)/S block in asynchronously growing cells and prevents G(1) entry in quiescent cells. Virology. 2000; 267:335–349. 10662629 10.1006/viro.1999.0147

[33] Daughenbaugh KF, Wobus CE, Hardy ME. VPg of murine norovirus binds translation initiation factors in infected cells. Virol J. 2006; 3:33 16719923 10.1186/1743-422X-3-33 PMC1481632

[34] Eskelin K, Hafren A, Rantalainen KI, Makinen K. Potyviral VPg enhances viral RNA Translation and inhibits reporter mRNA translation in planta. J Virol. 2011; 85:9210–9221. 10.1128/JVI.00052-11 21697470 PMC3165822

[35] Belliot G, Sosnovtsev SV, Chang KO, McPhie P, Green KY. Nucleotidylylation of the VPg protein of a human norovirus by its proteinase-polymerase precursor protein. Virology. 2008; 374:33–49. 10.1016/j.virol.2007.12.028 18234264 PMC2386983

[36] Goodfellow I. The genome-linked protein VPg of vertebrate viruses—a multifaceted protein. Curr Opin Virol. 2011; 1:355–362. 10.1016/j.coviro.2011.09.003 22440837 PMC3541522

[37] Leen EN, Kwok KY, Birtley JR, Simpson PJ, Subba-Reddy CV, Chaudhry Y, et al Structures of the compact helical core domains of feline calicivirus and murine norovirus VPg proteins. J Virol. 2013; 87:5318–5330. 10.1128/JVI.03151-12 23487472 PMC3648151

[38] Goodfellow I, Chaudhry Y, Gioldasi I, Gerondopoulos A, Natoni A, Labrie L, et al Calicivirus translation initiation requires an interaction between VPg and eIF 4 E. EMBO Rep. 2005; 6:968–972. 16142217 10.1038/sj.embor.7400510 PMC1369186

[39] Hosmillo M, Chaudhry Y, Kim DS, Goodfellow I, Cho KO. Sapovirus translation requires an interaction between VPg and the cap binding protein eIF4E. J Virol. 2014; 88:12213–12221. 10.1128/JVI.01650-14 25142584 PMC4248917

[40] Daughenbaugh KF, Fraser CS, Hershey JW, Hardy ME. The genome-linked protein VPg of the Norwalk virus binds eIF3, suggesting its role in translation initiation complex recruitment. EMBO J. 2003; 22:2852–2859. 12773399 10.1093/emboj/cdg251 PMC156748

[41] Beretta L, Svitkin YV, Sonenberg N. Rapamycin stimulates viral protein synthesis and augments the shutoff of host protein synthesis upon picornavirus infection. J Virol. 1996; 70:8993–8996. 8971030 10.1128/jvi.70.12.8993-8996.1996 PMC190998

[42] Chulu JL, Huang WR, Wang L, Shih WL, Liu HJ. Avian reovirus nonstructural protein p17-induced G(2)/M cell cycle arrest and host cellular protein translation shutoff involve activation of p53-dependent pathways. J Virol. 2010; 84:7683–7694. 10.1128/JVI.02604-09 20484520 PMC2897625

[43] Hyde JL, Mackenzie JM. Subcellular localization of the MNV-1 ORF1 proteins and their potential roles in the formation of the MNV-1 replication complex. Virology. 2010; 406:138–148. 10.1016/j.virol.2010.06.047 20674956

